# Supplementary material for: Nucleic acid mediated activation of a short prokaryotic Argonaute immune system
Source: Nat Commun. 2024 Jun 6;15:4852. doi: 10.1038/s41467-024-49271-4 (PMC11156904; doi:10.1038/s41467-024-49271-4)
Supplement: Supplementary file 1 — Supplementary Information [file 41467_2024_49271_MOESM1_ESM.pdf]

Supplementary Information

Nucleic Acid Mediated Activation of a Short Prokaryotic Argonaute Immune System

Jithesh Kottur[1,2*¶], Radhika Malik[1*¶], and Aneel K. Aggarwal[1¶]

[1]Department of Pharmacological Sciences, Icahn School of Medicine at Mount Sinai, New York, NY, 10029, USA

[2]Department of Antiviral Drug Research, Institute of Advanced Virology, Thiruvananthapuram, Kerala, 695317, India

[*] These authors contributed equally to this work

[¶]Corresponding authors:

E-mail: jitheshkottur@iav.res.in

E-mail: radhika.malik@mssm.edu

E-mail: aneel.aggarwal@mssm.edu

This file includes:

Supplementary Figures 1-11

Supplementary Table 1 and 2

Supplementary References

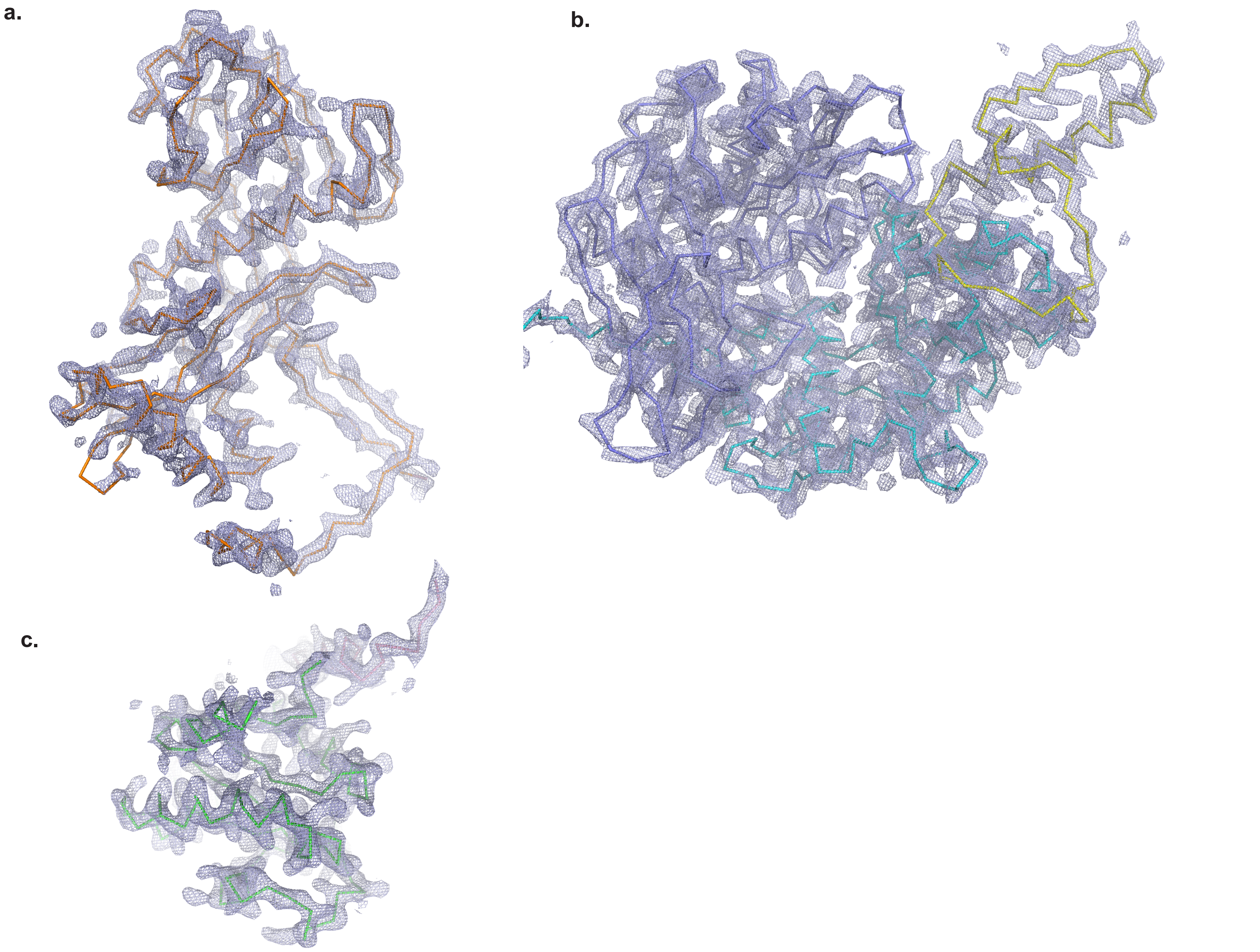

Supplementary Figure 1. Electron density maps of SPARTA domains. 2Fo-Fc maps of **(a)** APAZ, **(b)** Short pAgo and **(c)** TIR domains were shown at a contour level of 1.5σ. The individual domains are represented as ribbon form. APAZ domain is colored as orange, MID, Insert57 and PIWI domain are colored cyan, yellow and slate, respectively. The TIR domain and connector helix are colored as green and magenta respectivley.

a.

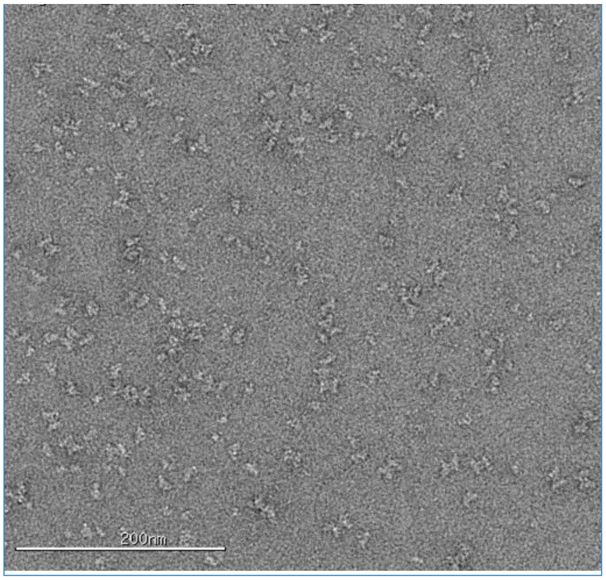

b.

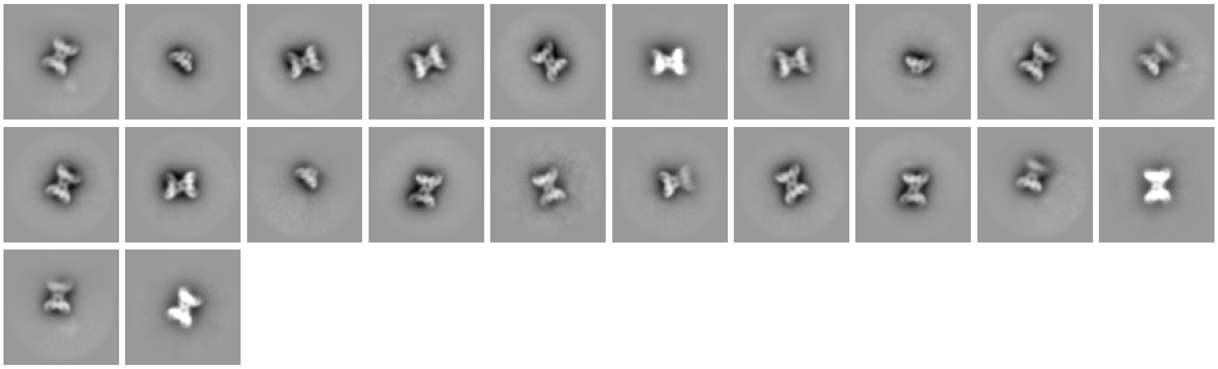

c.

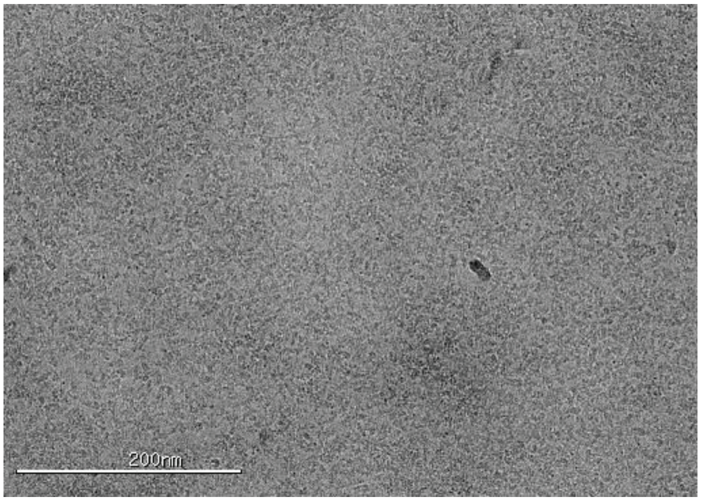

Supplementary Figure 2. Negative stain and cryo-electron microscopy . (a) Representative micrograph of a negatively stained sample of the SPARTA complex. Scale bar is 200 nm. The sample was stained with uranyl formate. (b) Representative 2-D classes after collect-ing data on negatively stained samples of the SPARTA oligomer (c) Representative micrograph of the tilted cryo-EM data of the SPARTA oligomer. This micrograph was imaged by tilting the stage to 30°.

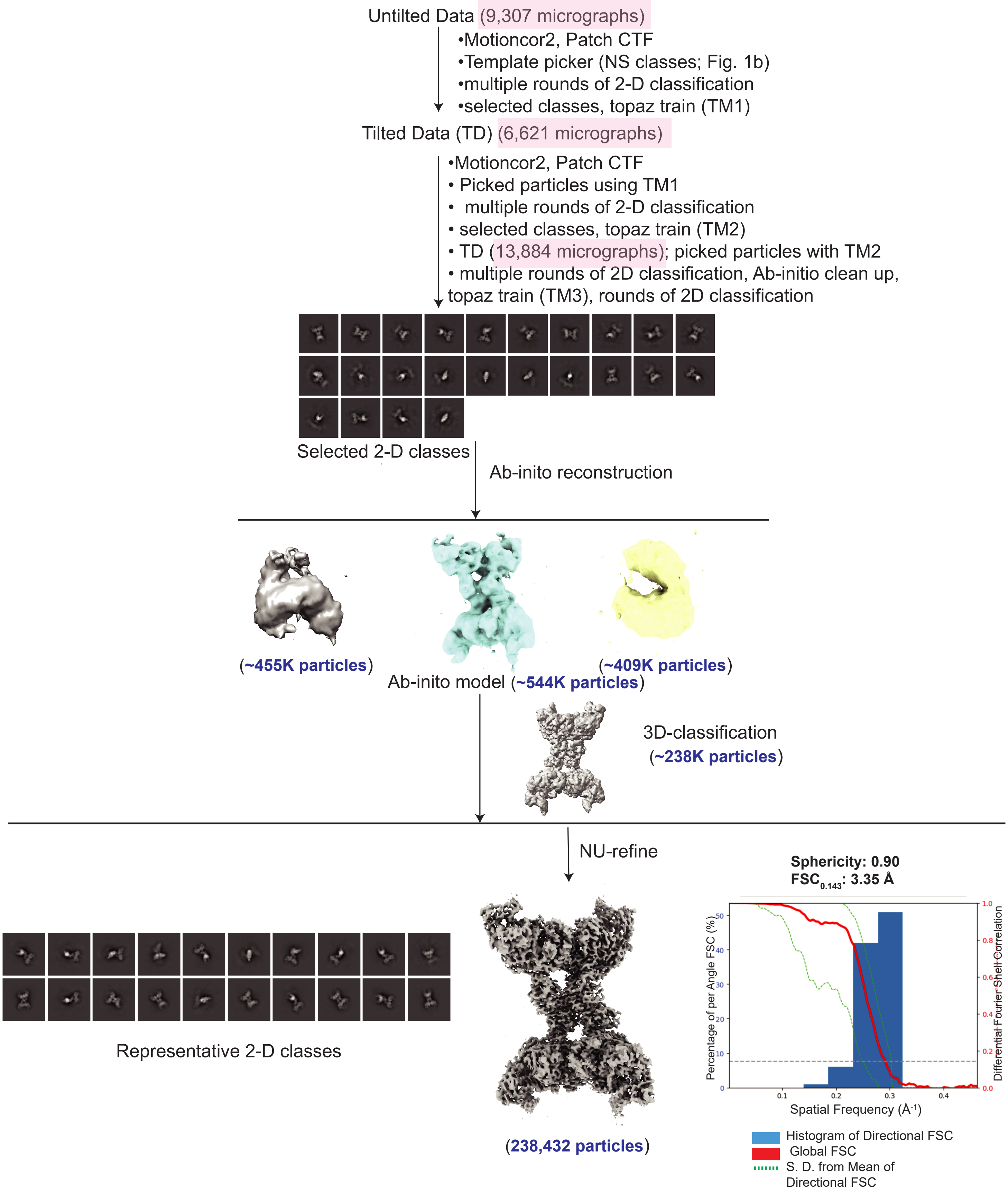

Supplementary Figure 3. Cryo-EM analysis of the SPARTA complex. An overview workflow of the cryo-EM image processing for the SPARTA complex is shown. Schematic shows particle picking using template picker and Topaz. Various stages of data processing includes 2-D classification, Ab-initio clean-up and 3-D classification using cryoSPARC. The number of micrographs collected is highlighted in pink. The number of particles at the various stages of data processing are shown in blue. The map calculated after global refinement has a $FSC_{0.143}$ of 3.35 Å. The 3D FSC plot to access the directional anisotropy is shown and the calculated sphericity is 0.90.

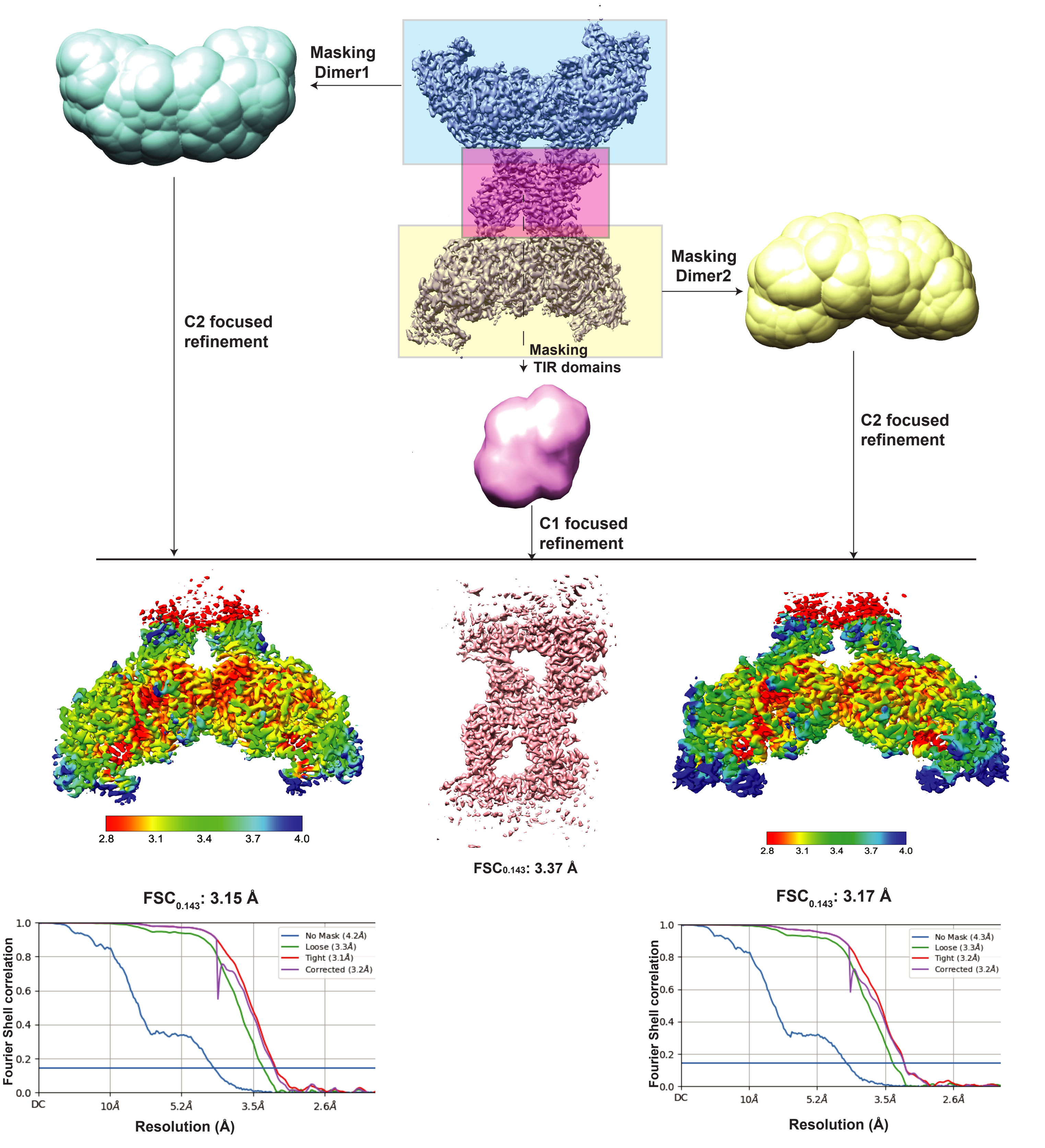

Supplementary Figure 4. Focused refinements of the SPARTA complex. A schematic workflow of the C2 symmetry based focused refinement of the 'wings' (APAZ-MID-PIWI-RNA/DNA) is described. Improvement in the resolution of the 'wings' is shown by the local resolution maps and their respective fourier shell correlation curves. FSC curves are shown and the nominal resolution of the focused maps for the 'wings' are 3.15 Å and 3.17 Å, respectively.

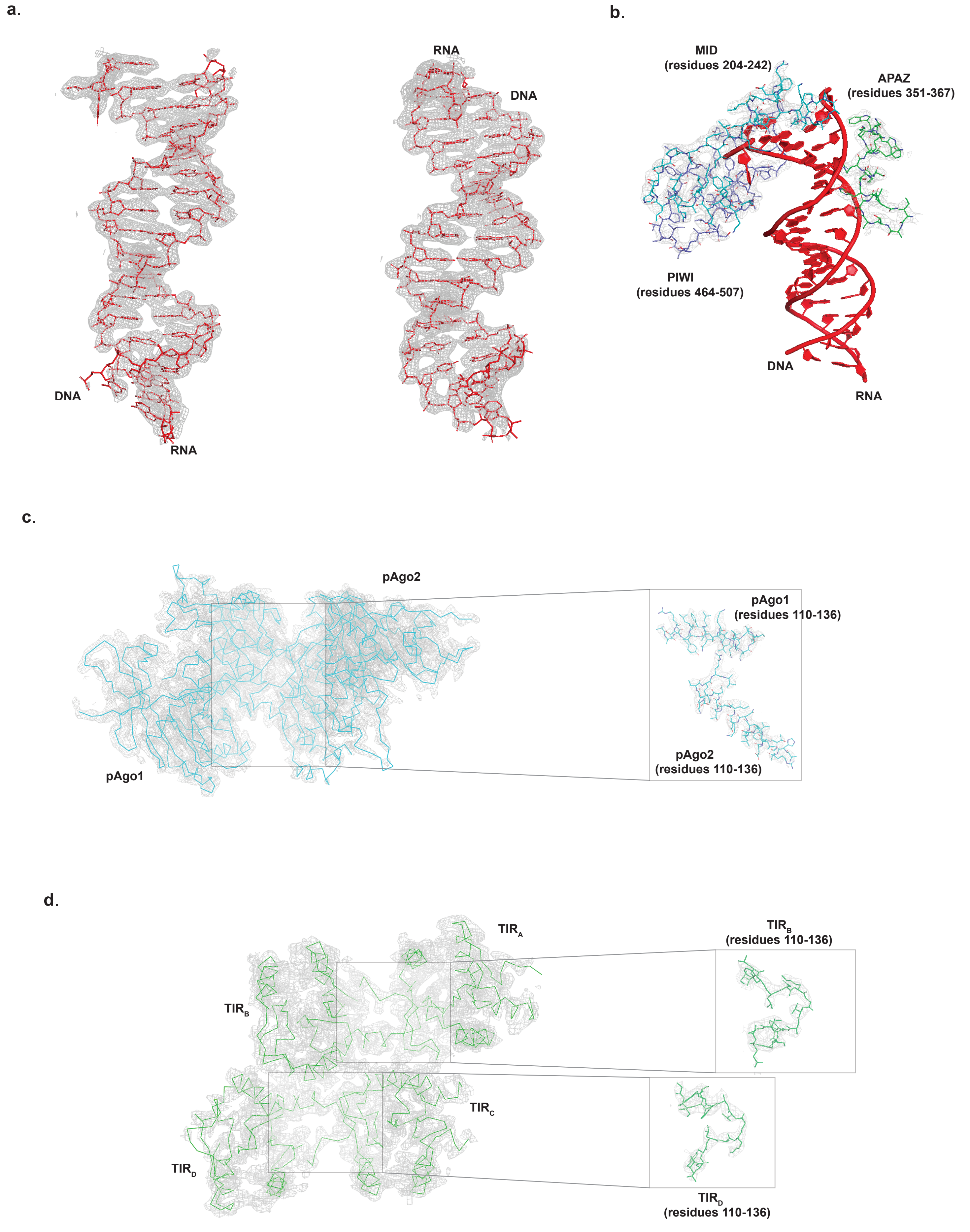

Supplementary Figure 5. Cryo-EM density for key regions of the SPARTA oligomer. (a) Cryo-EM density of the ligand, DNA/RNA duplex in two different orientations (90° apart) is shown. (b) Cryo-EM density of the key regions of MID (cyan) and PIWI (purple) domains of pAgo and APAZ (green) domain which are interacting with the RNA/DNA is depicted. (c) Cryo-EM density of one of the pAgo dimers and a close-up view of a section of the dimer interface is shown. (d) Cryo-EM density of all the TIR domains is shown and a section of the interface from TIR_B and TIR_D is shown in detail.

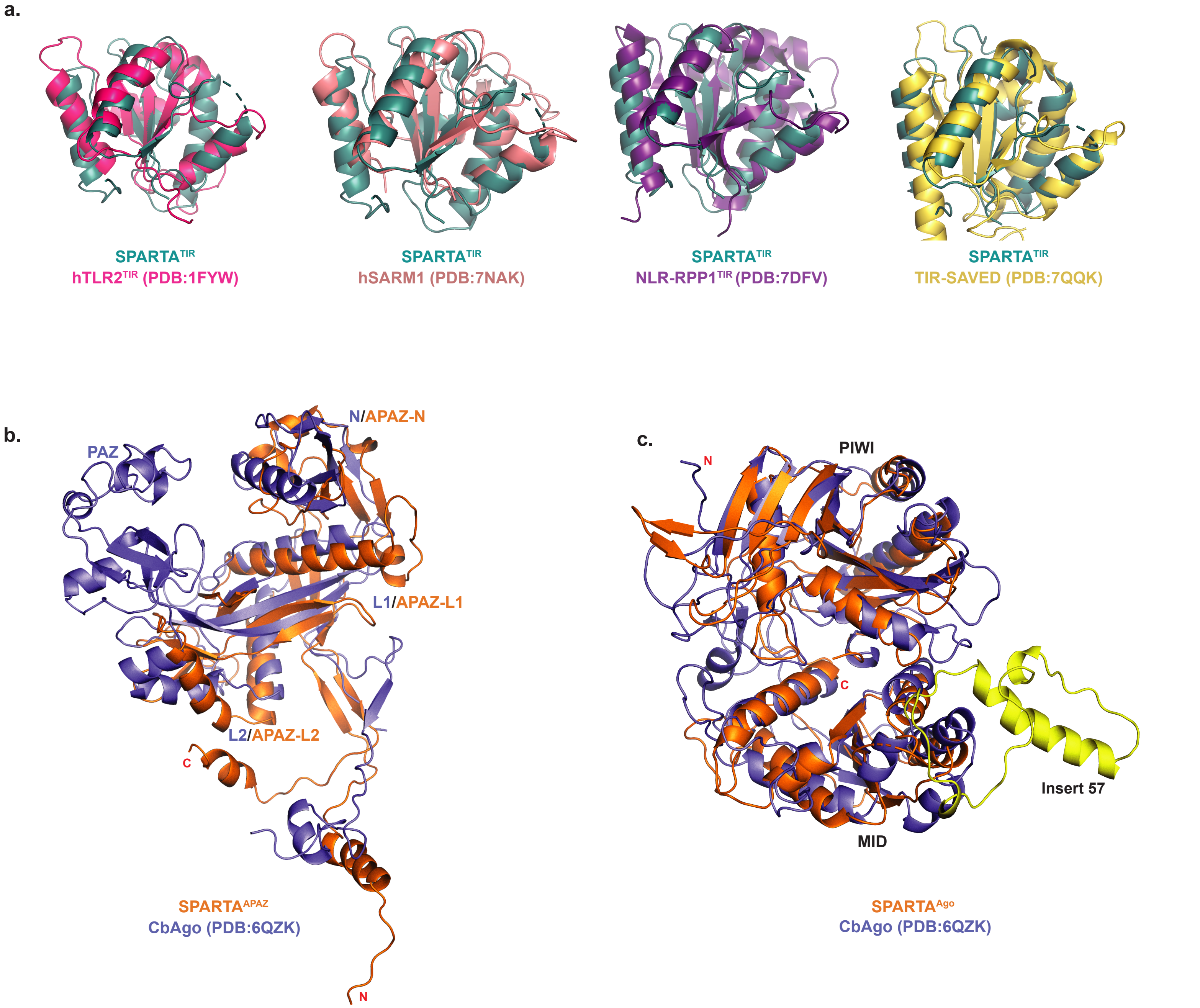

Supplementary Figure 6. Structural alignment of Apo SPARTA domains with homologous proteins. (a) SPARTA TIR domain structurally aligned to TIR domains from proteins with scaffolding roles (hTLR2^{TIR}, PDB:1FYW and TIR-SAVED, PDB:7QQK) and enzymatic roles (hSARM1, PDB:7NAK and NLR-RPP1^{TIR}, PDB:7DFV). (b) SPARTA APAZ domain structurally aligned with the N-PAZ-L1-L2 regions of *Clostridium butyricum* (Cb) long pAgo (PDB ID: 6QZK). (c) SPARTA MID and PIWI domains structurally aligned to the MID and PIWI domains of Cb long pAgo (PDB ID: 6QZK).

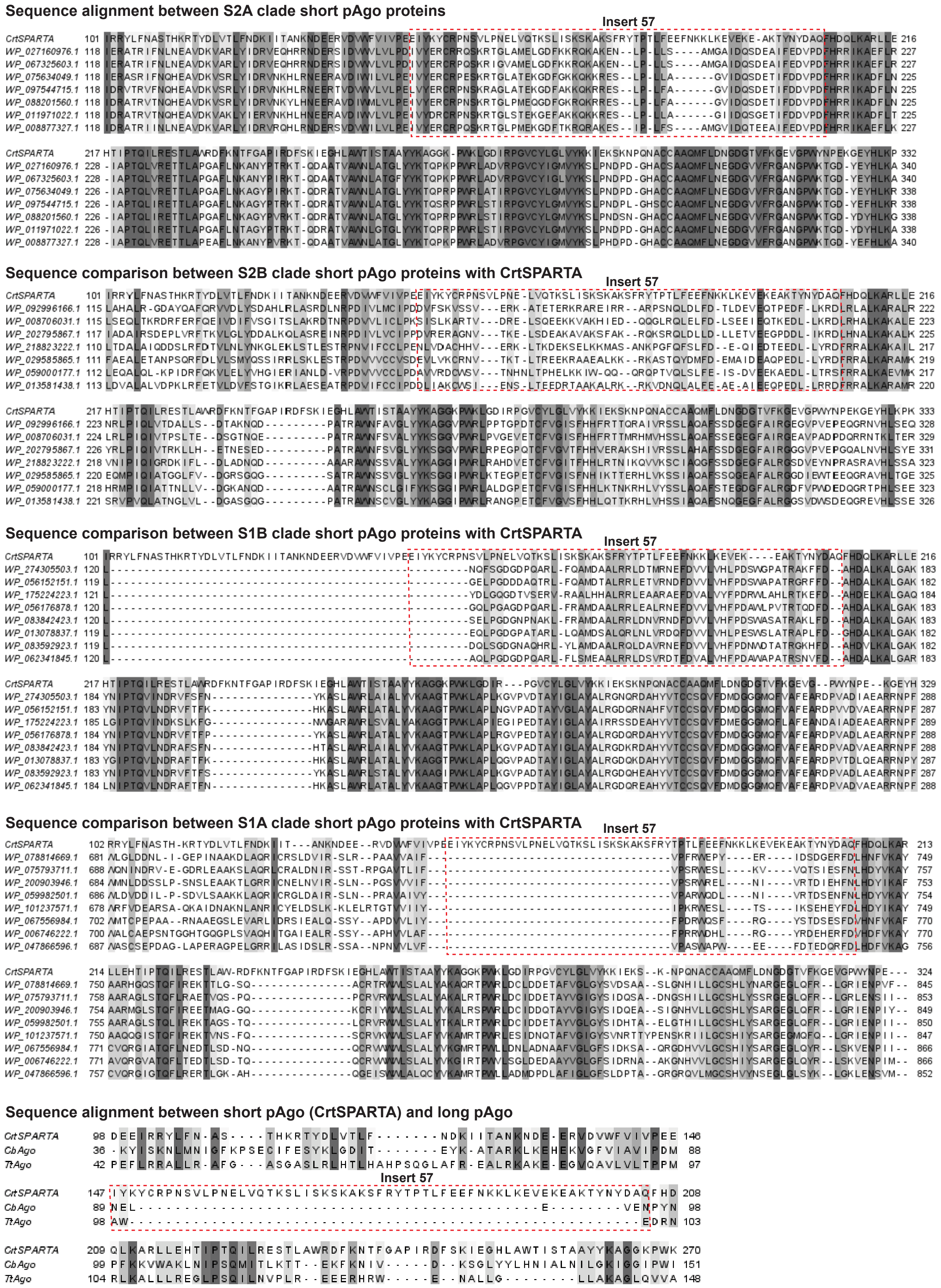
Sequence alignment between S2A clade short pAgo proteins
Insert 57
Sequence comparison between S2B clade short pAgo proteins with CrtSPARTA
Insert 57
Sequence comparison between S1B clade short pAgo proteins with CrtSPARTA
Insert 57
Sequence comparison between S1A clade short pAgo proteins with CrtSPARTA
Insert 57
Sequence alignment between short pAgo (CrtSPARTA) and long pAgo
Insert 57

Supplementary Figure 7. Sequence alignment of CrtSPARTA pAgo with short and long pAgos. CrtSPARTA pAgo (of clade S2A) aligned with all short pAgo clades (S2A, S2B, S1B, and S1A), as well as long pAgos (*Clostridium butyricum* CbAgo and *Thermus thermophilus* TtAgo). Insert57 in the short pAgo clades is highlighted in a dashed red box. Sequence alignments were conducted using Jalview software[1].

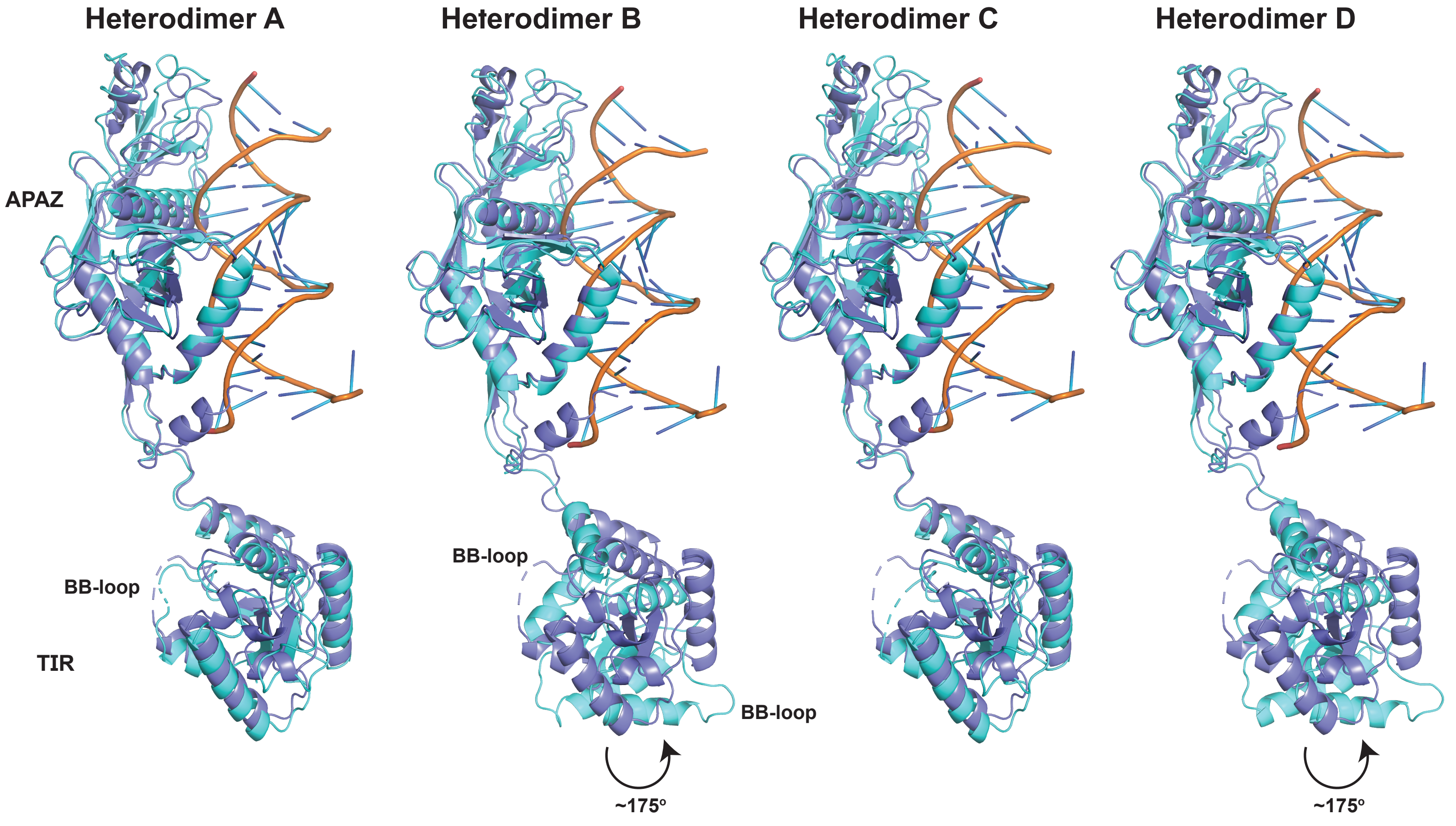

Supplementary Figure 8. Conformational changes of the TIR domain in the SPARTA heterodimers. Comparison of the TIR and APAZ domains in the inactive Apo SPARTA heterodimer versus the heterodimers A-D in the activated SPARTA oligomer. In the heterodimers B and D, the TIR domain undergoes ~175° rotation, as shown in the figure. The location of the BB-loop is marked to identify the conformational change.

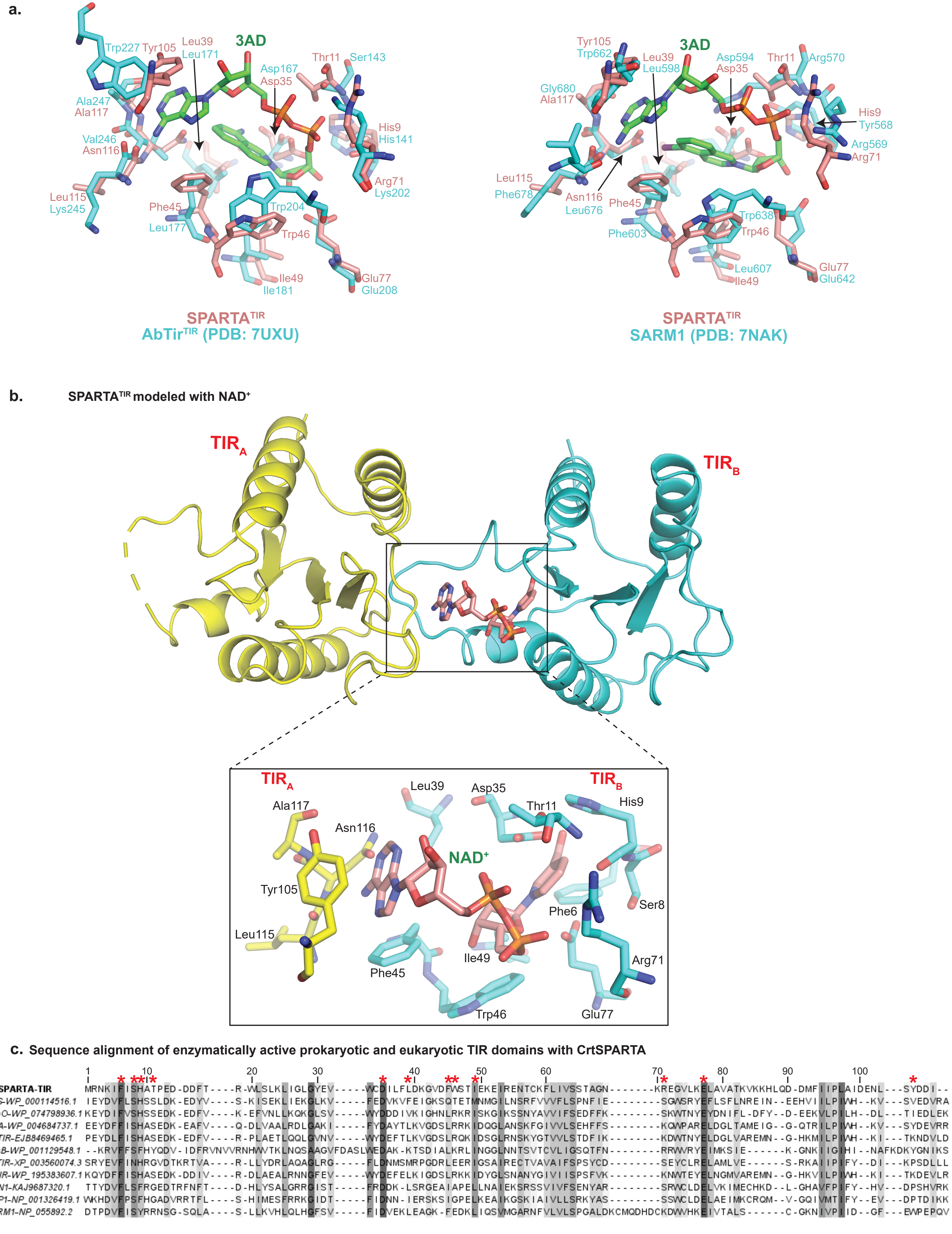

a.
3AD
Trp227 Tyr105
Leu39 Leu171
Asp167 Asp35
Thr11 Ser143
Ala247 Ala117
Val246 Asn116
His9 His141
Leu115 Lys245
Phe45 Leu177
Trp204 Trp46
Arg71 Lys202
Ile49 Ile181
Glu77 Glu208
SPARTA^TIR
AbTir^TIR (PDB: 7UXU)
Tyr105 Trp662
Leu39 Leu598
Asp594 Asp35
Thr11 Arg570
Gly680 Ala117
His9 Tyr568
Arg569 Arg71
Leu115 Phe678
Asn116 Leu676
Phe45 Phe603
Trp638 Trp46
Leu607 Ile49
Glu77 Glu642
SPARTA^TIR
SARM1 (PDB: 7NAK)
b. SPARTA^TIR modeled with NAD+
TIR_A
TIR_B
Leu39
Asp35
Thr11
His9
Ala117
Asn116
NAD+
Tyr105
Ser8
Phe6
Leu115
Ile49
Arg71
Phe45
Trp46
Glu77
c. Sequence alignment of enzymatically active prokaryotic and eukaryotic TIR domains with CrtSPARTA
1 10 20 30 40 50 60 70 80 90 100
CrtSPARTA-TIR MRNKIFISHATPED-DDFT----R-WLSLKLIGLGYEV-----WCDILFLDKGVDFWSTIEKEIRENTCKFLIVSSTAGN--------KREGVLKELAVATKVKKHLQD-DMFIIPLAIDENL---SYDDI--
TirS-WP_000114516.1 IEYDVFLSHSSLDK-EDYV----S-KISEKLIEKGLKV-----FEDVKVFEIGKSQTETMNMGILNSRFVVVFLSPNFIE---------SGWSRYEFLSFLNREIN--EEHVIILPIWH--KV---SVEDVRA
TcpO-WP_074798936.1 KEYDIFVSHSSEDK-EDFV----K-EFVNLLKQKGLSV-----WYDDDIVKIGHNLRKRISKGIKSSNYAVVIFSEDFFK---------SKWTNYEYDNIFL-DFY--D-EEKVLPILH--DL---TIEDLEK
BtpA-WP_004684737.1 EEYDFFISHASEDK-EAFV----Q-DLVAALRDLGAKI-----FYDAYTLKVGDSLRRKIDQGLANSKFGIVVLSEHFFS---------KQWPARELDGLTAMEIG--G-QTRILPIWH--KV---SYDEVRR
AbTIR-EJB8469465.1 PEYDLFISHASEDK-EDFV----R-PLAETLQQLGVNV-----WYDEFTLKVGDSLRQKIDSGLRNSKYGTVVLSTDFIK---------KDWTNYELDGLVAREMN--G-HKMILPIWH--KI---TKNDVLD
ThsB-WP_001129548.1 --KRVFFSFHYQDV-IDFRVNVVRNHWVTKLNQSAAGVFDASLWEDAK-KTSDIALKRLINGGLNNTSVTCVLIGSQTFN---------RRWVRYEIMKSIE------K-GNKIIGIHI--NAFKDKYGNIKS
BdTIR-XP_003560074.3 SRYEVFINHRGVDTKRTVA----R-LLYDRLAQAGLRG-----FLDNMSMRPGDRLEERIGSAIRECTVAVAIFSPSYCD---------SEYCLRELAMLVE------S-RKAIIPIFY--DI---KPSDLLL
BtTIR-WP_195383607.1 KQYDFFISHASEDK-DDIV----R-DLAEALRNNGFEV-----WYDEFELKIGDSLRKKIDYGLSNANYGIVIISPSFVK---------KNWTEYELNGMVAREMN--G-HKVILPIWH--KI---TKDEVLR
RUN1-KAJ9687320.1 TTYDVFLSFRGEDTRFNFT----D-HLYSALGRRGIST-----FRDDK-LSRGEAIAPELLNAIEKSRSSVIVFSENYAR---------SRWCLDELVKIMECHKD--L-GHAVFPIFY--HV---DPSHVRK
RPP1-NP_001326419.1 WKHDVFPSFHGADVRRTFL----S-HIMESFRRKGIDT-----FIDNN-IERSKSIGPELKEAIKGSKIAIVLLSRKYAS---------SSWCLDELAEIMKCRQM--V-GQIVMTIFY--EV---DPTDIKK
SARM1-NP_055892.2 DTPDVFISYRRNSG-SQLA----S-LLKVHLQLHGFSV-----FIDVEKLEAGK-FEDKLIQSVMGARNFVLVLSPGALDKCMQDHDCKDWVHKEIVTALS------C-GKNIVPIID--GF---EWPEPQV

Supplementary Figure 9. Comparison of TIR active sites and modeling of NAD⁺. (a) Structural superposition of SPARTA active site formed across the TIR$_A$ and TIR$_B$ heterodimers with that in AbTir (PDB:7UXU) (left) and SARM1 (PDB: 7NAK) (right) containing bound 3AD. (b) TIR domains of SPARTA heterodimers modeled with NAD⁺ and a close-up view of the active site showing the putative residues interacting with NAD⁺. (c) Sequence alignment of enzymatically active prokaryotic and eukaryotic TIR domains with SPARTA^{TIR}. The active site residues are highlighted with asterisks (*) and show conservation among members of enzymatically active TIR domains from bacterial (TirS, TcpO, BtpA, AbTIR, ThsB, BdTIR, BtTIR), plant (RUN1, RPP1), and human (SARM1). Sequence alignments were carried out using Jalview software[1].

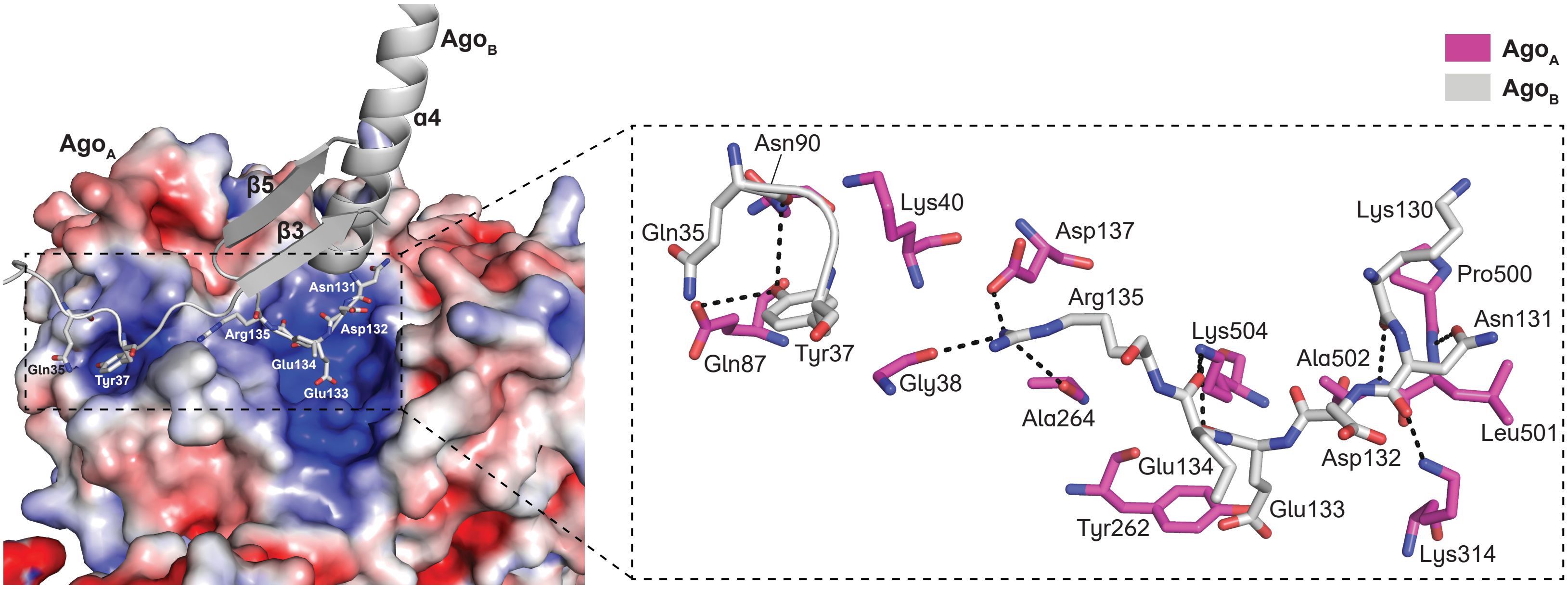

Supplementary Figure 10. Structural basis for pAgo-mediated oligomerization. Close-up view of the pAgo dimer interface showing a positively charged pocket on pAgo$_A$ receiving residues from pAgo$_B$. Residues Gln35 and Tyr37 of pAgo$_B$ are involved in additional interactions with pAgo$_A$.

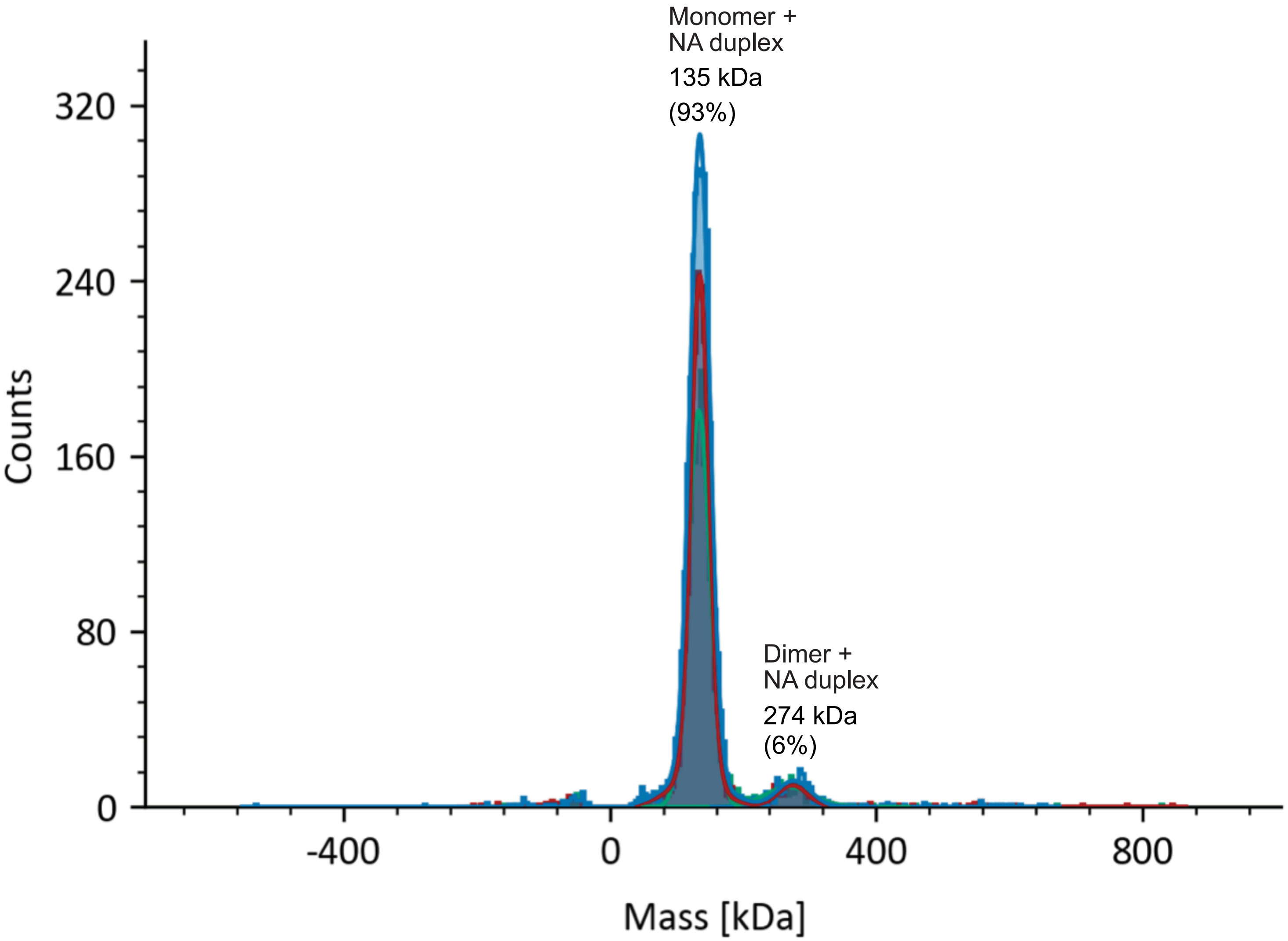

Supplementary Figure 11. Mass photometry data for the SPARTA deletion mutant. Mass photometry data for the SPARTA deletion mutant, in which residues from the loop10-9 were deleted (pAGOΔAA322-327), incubated with guide RNA (gRNA) and target DNA (tDNA). The mass distribution plot shows that the majority of the population (~93%) is monomeric SPARTA bound to the gRNA/tDNA, with an average MW of ~134.6 kDa (close to the expected MW of 132.6 kDa) with only a very small fraction forming SPARTA dimeric-gRNA/tDNA complexes (~6%), with an average MW of ~273 kDa (close to the expected MW of 265.2 kDa) .

Supplementary Table 1. X-ray data collection and refinement statistics for Apo-SPARTA

	Apo-SPARTA **(PDB: 8U7B)**
**Data collection**	
Space group	$P6_522$
Cell dimensions	
a, *b*, *c* (Å)	197.18 Å, 197.18 Å 183.4 Å,
α, β, γ (°)	90.00 90.00 120.00
Resolution (Å)	170.76-2.66 (2.99-2.66)*
R_{sym} or R_{merge}	14.8 (176.7)
R_{pim}	4.2 (55.4)
No. of unique reflections	40914
$I / \sigma I$	13.7 (1.7)
Completeness (%) (ellipsoidal)	96.2 (73.9)
Redundancy	13.3 (10.7)
CC(1/2)	1.00 (0.57)
**Refinement**	
Resolution (Å)	49.71-2.66
No. reflections	40895
R_{work} / R_{free}	18.6/24.0
No. atoms	
Macromolecules	7420
Ligands/Ions	2
Water	91
B-factors	
Macromolecules	69.0
Ligands/Ions	67.0
Water	63.9
R.m.s. deviations	
Bond lengths (Å)	0.003
Bond angles (°)	0.61
Ramachandran Plot	
Favored (%)	93.0
Allowed (%)	6.5
Outliers (%)	0.5

Supplementary Table 2. CryoEM data collection, model refinement and validation statistics

	SPARTA-gRNA-tDNA-Mg^{2+}<br>**PDB ID:8U72**<br>**EMDB IDs: EMD-41945, EMD-41947, EMD-41948, EMD-41959, EMD-41966**
**Data collection and processing**	
Magnification (kV)	x81000
Voltage (kV)	300
Pixel Size (Å/pixel)	1.083
Electron dose (e^-/Å^2)	50.79
Defocus range (μm)	0.8 - 2.5
Number of Micrographs	13,884
Tilt angle	30º, 45º
Number of Particles	238,432
Nominal Map resolution (Å)	3.35
FSC threshold	0.143
**Refinement (Phenix)**	
**Model composition**	
Non-hydrogen atoms	29715
Protein residues	3469
DNA/other	148/4
**R.M.S. deviations**	
Bond lengths (Å)	0.003
Bond Angles (°)	0.596
**Validation**	
Molprobity score	1.90
Clashscore	7.07
Rotamer outliers (%)	0.33
Cβ outliers (%)	0.00
**Ramachandran Statistics (%)**	
Favored	91.16
Allowed	8.78
Outliers	0.06

Supplementary References.

1. Waterhouse, A. M., Procter, J. B., Martin, D. M., Clamp, M. & Barton, G. J. Jalview Version 2-a multiple sequence alignment editor and analysis workbench. *Bioinformatics* **25**, 1189-1191 (2009).
