# Supplementary material for: Nucleic acid mediated activation of a short prokaryotic Argonaute immune system
Source: Nat Commun. 2024 Jun 6;15:4852. doi: 10.1038/s41467-024-49271-4 (PMC11156904; doi:10.1038/s41467-024-49271-4)
Supplement: Supplementary file 3 — Reporting Summary [file 41467_2024_49271_MOESM3_ESM.pdf]

nature portfolio

Corresponding author(s): Dr. Aneel K. Aggarwal

Last updated by author(s): May 30, 2024

Reporting Summary

Nature Portfolio wishes to improve the reproducibility of the work that we publish. This form provides structure for consistency and transparency in reporting. For further information on Nature Portfolio policies, see our Editorial Policies and the Editorial Policy Checklist.

Statistics

For all statistical analyses, confirm that the following items are present in the figure legend, table legend, main text, or Methods section.

n/a	Confirmed	
☐	☒	The exact sample size (*n*) for each experimental group/condition, given as a discrete number and unit of measurement
☐	☒	A statement on whether measurements were taken from distinct samples or whether the same sample was measured repeatedly
☒	☐	The statistical test(s) used AND whether they are one- or two-sided<br>*Only common tests should be described solely by name; describe more complex techniques in the Methods section.*
☒	☐	A description of all covariates tested
☒	☐	A description of any assumptions or corrections, such as tests of normality and adjustment for multiple comparisons
☐	☒	A full description of the statistical parameters including central tendency (e.g. means) or other basic estimates (e.g. regression coefficient) AND variation (e.g. standard deviation) or associated estimates of uncertainty (e.g. confidence intervals)
☒	☐	For null hypothesis testing, the test statistic (e.g. *F*, *t*, *r*) with confidence intervals, effect sizes, degrees of freedom and *P* value noted<br>*Give P values as exact values whenever suitable.*
☒	☐	For Bayesian analysis, information on the choice of priors and Markov chain Monte Carlo settings
☒	☐	For hierarchical and complex designs, identification of the appropriate level for tests and full reporting of outcomes
☒	☐	Estimates of effect sizes (e.g. Cohen's *d*, Pearson's *r*), indicating how they were calculated

Our web collection on statistics for biologists contains articles on many of the points above.

Software and code

Policy information about availability of computer code

Data collection	X-ray diffraction data were collected at the NSLS-II 17-ID-1 and 17-ID-2 beamlines at the Brookhaven National Laboratory (BNL) under cryogenic conditions. Mass photometry experiments and most of the EM work was performed at the Simons Electron Microscopy Center and National Resource for Automated Molecular Microscopy, located at the New York Structural Biology Center.
Data analysis	The diffraction data were processed using autoPROC and STARANISO (Global Phasing Ltd.). The structure was solved by molecular replacement using Phaser-MR with a model of CrtSPARTA generated by AlphaFold as the search model. Subsequently, iterative manual building and refinement were performed using Coot v0.8.9.1 EL and Phenix v1.20cr3-4406-000 Refine. All molecular graphic figures were prepared using PyMOL v2.5.4 (Schrödinger LLC). Jalview v2.11.3.2 software was used for the sequence alignements.<br>For CryoEM:<br>Patch CTF within cryoSPARC v3.3.1 was used for CTF determination<br>MotionCor2 was used for aligning cryoEM images<br>Blob picker and Topazv0.2.3 within cryoSPARC v3.3.1 was used for picking particles from EM images<br>cryoSPARC v3.3.1 were used for 2D classification, 3D classification, EM map calculation, and symmetry based focused refinements.<br>COOT v0.9.3 was used for building structure models into the cryoEM map<br>PHENIX v1.20 was used for real space refinement of structure against the cryoEM map<br>UCSF ChimeraX v1.335 and PyMOL v2.5.4 were used for analyzing structures and generating figures.

For manuscripts utilizing custom algorithms or software that are central to the research but not yet described in published literature, software must be made available to editors and reviewers. We strongly encourage code deposition in a community repository (e.g. GitHub). See the Nature Portfolio guidelines for submitting code & software for further information.

Data

Policy information about availability of data

All manuscripts must include a data availability statement. This statement should provide the following information, where applicable:

- Accession codes, unique identifiers, or web links for publicly available datasets
- A description of any restrictions on data availability
- For clinical datasets or third party data, please ensure that the statement adheres to our policy

The structure factors and coordinate files for the crystal structure of the Apo SPARTA has been deposited in the Protein Data Bank (PDB) under the accession code 8U7B [http://doi.org/10.2210/pdb8U7B/pdb].
The original, composite cryo-EM density map as well as the focused refinement maps of the SPARTA oligomer generated in this study have been deposited in the Electron Microscopy Data Bank (EMDB) under accession numbers EMD-41959 [https://www.ebi.ac.uk/emdb/EMD-41959], (Original map), EMD-41945 [https://www.ebi.ac.uk/emdb/EMD-41945] (Focused refined map of the pAgo-APAZ dimer), EMD-41947 [https://www.ebi.ac.uk/emdb/EMD-41947] (Focused refined map of the second pAgo-APAZ dimer), EMD-41948 [https://www.ebi.ac.uk/emdb/EMD-41948] (Focused refined map of the TIR domains) and EMD-41966 [https://www.ebi.ac.uk/emdb/EMD-41966] (Composite map). The resulting atomic coordinates for the SPARTA oligomer have been deposited in the Protein Data Bank (PDB) with accession number PDB ID: 8U72 [http://doi.org/10.2210/pdb8U72/pdb].

Raw uncropped gel image (for fig. 1b) and raw ε-NAD+ assay data (for fig. 5b) are available as source data file accompanying this manuscript.

Research involving human participants, their data, or biological material

Policy information about studies with human participants or human data. See also policy information about sex, gender (identity/presentation), and sexual orientation and race, ethnicity and racism.

Reporting on sex and gender	NA
Reporting on race, ethnicity, or other socially relevant groupings	NA
Population characteristics	NA
Recruitment	NA
Ethics oversight	NA

Note that full information on the approval of the study protocol must also be provided in the manuscript.

Field-specific reporting

Please select the one below that is the best fit for your research. If you are not sure, read the appropriate sections before making your selection.

☒ Life sciences ☐ Behavioural & social sciences ☐ Ecological, evolutionary & environmental sciences

For a reference copy of the document with all sections, see nature.com/documents/nr-reporting-summary-flat.pdf

Life sciences study design

All studies must disclose on these points even when the disclosure is negative.

Sample size	The number of independent experiments and biological replicates was indicated in the relevant figure legends.
Data exclusions	No data were excluded from analysis
Replication	For the X-ray sructure reported in this manuscript, data sets were collected from more than 10 different crystals. The single data set with best diffraction quality were used for further structure solution and refinement.<br>For the cryo-EM maps, Fourier Shell correlation value of 0.143 between independently refined half sets was used to estimate the resolution of the map.<br>For the ε-NADase assay, the assay was done in triplicates and the mean value is reported.
Randomization	For the X-ray data, 5% data is randomly selected during structure refinement for cross validation.
Blinding	Blinding is not applicable to structural studies performed in this work. Also, blinding was not relevant in other experiments as the investigators need to be aware of wild type SPARTA and SPARTA mutants. For the cryo-EM maps, the nominal resolution was calculated using gold-standard Fourier Shell correlation.

Reporting for specific materials, systems and methods

We require information from authors about some types of materials, experimental systems and methods used in many studies. Here, indicate whether each material, system or method listed is relevant to your study. If you are not sure if a list item applies to your research, read the appropriate section before selecting a response.

Materials & experimental systems

n/a	Involved in the study	
☒	☐	Antibodies
☒	☐	Eukaryotic cell lines
☒	☐	Palaeontology and archaeology
☒	☐	Animals and other organisms
☒	☐	Clinical data
☒	☐	Dual use research of concern
☒	☐	Plants

Methods

n/a	Involved in the study	
☒	☐	ChIP-seq
☒	☐	Flow cytometry
☒	☐	MRI-based neuroimaging
